# NAD+ Boosting Through NRH Supplementation Enhances Treatment Efficacy in EOC In Vitro

**DOI:** 10.3390/ijms26041719

**Published:** 2025-02-18

**Authors:** Kevin J. Lee, Sagar Chokshi, Tanvi Joshi, Mackenzie Cummings, Catherine E. Lyons, Mary Howard Singleton, Elizabeth Catranis, Luciana Madiera da Silva, Faisal Hayat, Marie Migaud, Jennifer Scalici

**Affiliations:** 1 USA Health Mitchell Cancer Institute, University of South Alabama, Mobile, AL 36604, USA; 2 Department of Pharmacology, University of South Alabama, Mobile, AL 36688, USA; 3 Winship Cancer Institute, Emory University School of Medicine, Atlanta, GA 30322, USA

**Keywords:** NRH, NAD+, ovarian cancer, PARP inhibitors, olaparib

## Abstract

Dihydronicotinamide rioside (NRH), the reduced form of nicotinamide riboside (NR), is a recently identified, naturally occurring precursor of arguably the most crucial cofactor for cellular function, nicotinamide adenine dinucleotide (NAD+). Recent investigation suggests that NRH is more adept at increasing NAD+ stores than traditional NAD+ precursors, and such extreme NAD+ boosting via NRH supplementation induces cytotoxicity in certain cellular contexts. It has also been shown that the lack of functional BRCA protein in epithelial ovarian cancer (EOC) directly impacts intracellular NAD+ levels. Given that altered cellular metabolism and DNA repair mechanisms are central alterations in EOC, and these processes are functionally dependent on NAD+, we sought to assess whether NRH supplementation in EOC cell lines enhanced cellular cytotoxicity alone and in combination with standard therapeutic agents. Significant cytotoxicity was noted in NRH treated cells (~40%) with minimal cell death in the nicotinic acid (NA)-treated lines. Levels of NAD(P)H were confirmed to have increased with NRH supplementation, albeit at different levels among the different cell lines. Overall, the cytotoxicity associated with NRH supplementation appears to be independent of ROS generation. Strikingly, NRH supplementation enhanced cytotoxicity of carboplatin in OVCAR8, but not ES2 or SKOV3. Paclitaxel cytotoxicity was also enhanced by the addition of NRH in OVCAR8, but not ES2 or SKOV3 cell lines. NA supplementation had no effect on baseline treatment-induced cytotoxicity. PARP inhibition by olaparib requires NAD+. Interestingly, NRH supplementation enhanced olaparib cytotoxicity in SKOV3 and OVCAR8, but not ES2 cells. NRH in combination with olaparib completely altered mitochondrial respiration, thereby shutting down energy consumption, which would lead to cell death. Coupled together with expression data of key enzymes required for NRH/NAD metabolism, this could be key in understanding mechanisms of cell death with NRH supplementation. Here, we showed that in the context of EOC, exploitation of the NAD+ bioenergetic phenotype through NRH supplementation is a biologically feasible strategy to enhance the response of traditional therapy with potentially minimal toxicity. These data suggest several potential mechanisms by which cellular NAD+ availability impacts treatment efficacy and resistance and highlights the potential utility of NAD+ metabolomics as a biomarker to guide treatment decisions.

## 1. Introduction

A shift in cellular metabolism is a known hallmark in the development of cancer, as tumor cells shift their biochemical phenotype to support their increasing metabolic demand [1]. Nicotinamide adenine dinucleotide (NAD+) is an important coenzyme and substrate that mediates reactions in multiple metabolic pathways, including DNA damage repair, antioxidant balance, and bioenergy production [2,3,4]. Unsurprisingly, this molecule is thought to underlie many aspects of aging and disease development, including carcinogenesis [4,5,6,7]. The relationship between NAD+ and cancer development is multifaceted and at times unclear due to the complex nature of NAD+ metabolism [5].

Many cellular signaling mechanisms use NAD+ for enzymatic processes such as those enabled by Poly (ADP-ribose) polymerases (PARP) [8]. PARP is one of the key enzymes involved in DNA damage repair that plays a role in multiple pathways of repair, including base excision repair, single-strand repair, double-strand repair, and bulky lesion repair among others [9]. PARP uses NAD+ in the generation of poly (ADP-ribose) units (PAR) as a chain coming off PARP in order to recruit other DNA repair proteins for functional repair [10]. PARP inhibitors have become one of the mainstays of treatment for several tumor types, including ovarian cancer, through the synthetic lethality of inherent aberrancies in DNA damage/repair mechanisms and inhibition of the fundamental function of PARP [11,12]. Despite tremendous advances in the use of PARP inhibitors, ovarian cancer remains the most lethal gynecologic malignancy [13]. PARP inhibition following DNA-damaging, platinum-based chemotherapy has improved overall survival in some ovarian cancer patients, especially those with mutations in breast cancer-associated genes 1 and 2 (BRCA1/2), DNA damage repair genes, or defects in homologous recombination. However, this response is not seen in all patients and resistance to these agents remains an issue [11,14]. One limitation is thought to be the metabolic and bioenergetic adaptations that cells undergo in a low NAD+ microenvironment [2,7]. The work presented here showed that our group had identified a potential role for NAD+ boosting to enhance the efficacy of ovarian cancer treatment in an in vitro model.

As NAD+ is generated from vitamin B3 derivatives, including niacinamide (nicotinamide, Nam), nicotinic acid (NA), nicotinamide riboside (NR), nicotinamide mononucleotide (NMN), and tryptophan, it is feasible that a metabolic approach to enhancing ovarian cancer treatment could be dietary in nature. Dihydronicotinamide riboside (NRH), the reduced form of nicotinamide riboside (NR), is a recently identified, naturally occurring precursor of NAD+ [15,16]. Recent investigation suggested that NRH is more adept at increasing NAD+ stores than traditional NAD+ precursors, and such extreme NAD+ boosting via NRH supplementation induces cytotoxicity in certain cellular contexts [16,17]. To this end, we sought to assess whether NRH supplementation in epithelial ovarian cancer (EOC) cell lines enhanced cellular cytotoxicity alone and in combination with standard therapeutic agents such as the PARP inhibitor, olaparib.

## 2. Results

**Figure 1:**
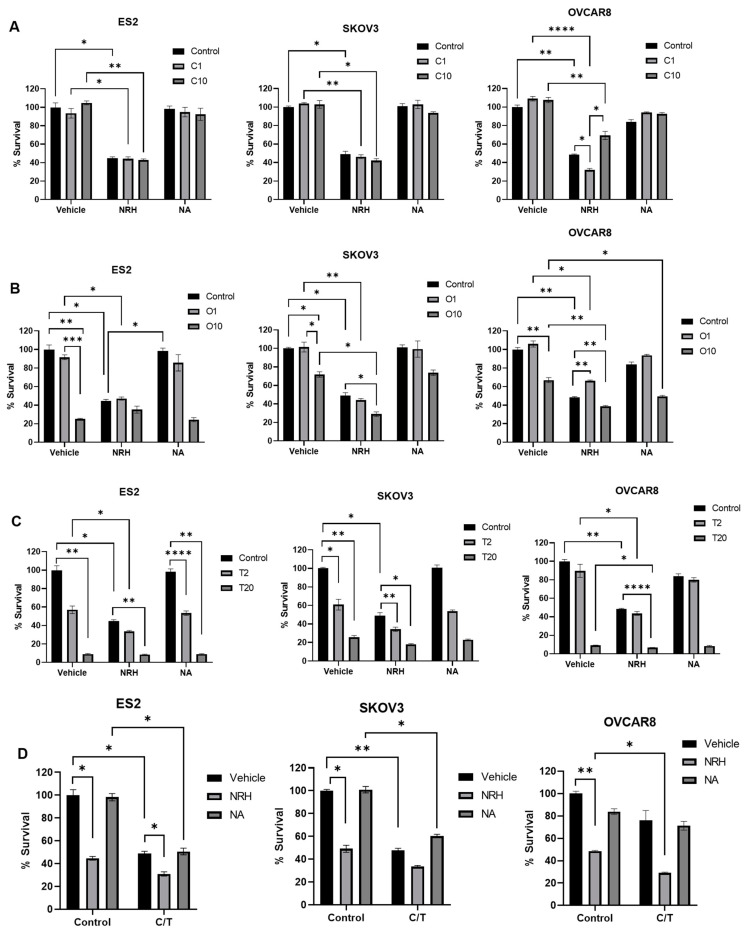
Cytotoxicity of chemotherapeutic agents in combination with NRH. (**A**) EOC cell lines were treated with control, 1 µM carboplatin (C1), or 10 µM carboplatin (C10) alone or in combination with NRH (500 µM) or NA (50 µM). (**B**) EOC cell lines were treated with control, 1 µM olaparib (O1), or 10 µM olaparib (O10) alone or in combination with NRH (500 µM) or NA (50 µM). (**C**) EOC cell lines were treated with control, 2 nM paclitaxel (T2), or 20 nM paclitaxel (T20) alone or in combination with NRH (500 µM) or NA (50 µM). (**D**) EOC cell lines were treated with control or 1 µM carboplatin + 2 nM paclitaxel (C/T) alone or in combination with NRH (500 µM) or NA (50 µM). * *p* < 0.05; ** *p* < 0.01; *** *p* < 0.001; **** *p* < 0.0001.

Ovarian cancer cell lines were treated with standard first-line ovarian cancer therapies: carboplatin (Figure 1A), olaparib (Figure 1B), paclitaxel (Figure 1C), or carboplatin + paclitaxel (C/T) (Figure 1D); in the presence of either chemotherapeutic alone or in combination of either NRH or NA for 72 h. As seen in Figure 1A, NRH alone induces cytotoxicity in all three cell lines tested at roughly the same rate (ES2-55% cell death, SKOV3-51% cell death, and OVCAR8-52% cell death). Although carboplatin + NRH also shows significant cytotoxicity compared to vehicle, there was no difference compared to NRH alone in either ES2 or SKOV3. However, low-dose carboplatin (1 µM) showed a significant combination effect in OVCAR8 compared to both carboplatin alone and to NRH alone with 68% cell death.

Interestingly, as seen in Figure 1B, olaparib at 10 µM was highly cytotoxic in ES2 cells (~70–75% cell death), yet no combination effect was seen with NRH. In contrast, SKOV3 showed a significant combination effect with olaparib 10 µM + NRH (71% cell death) compared with olaparib alone (28% cell death) as well as NRH alone (51% cell death). OVCAR8 showed similar results with a significant combination effect of olaparib 10 µM + NRH, resulting in 61% cell death compared to olaparib alone (33% cell death) and NRH alone (52% cell death).

As shown in Figure 1C, high-dose paclitaxel (20 nM) proved to be highly cytotoxic, resulting in roughly 80–90% cell death in all cells tested. Therefore, determination of combination effects with this dose is inconclusive. ES2 cells showed significantly greater cytotoxicity with the combination of paclitaxel 2 nM + NRH (67% cell death) than paclitaxel alone (43% cell death). Interestingly, the combination was not statistically more cytotoxic than NRH alone (55% cell death). Again, in contrast to the ES2 line, SKOV3 cells exhibited significantly more cytotoxicity with the combination of paclitaxel 2 nM + NRH (66% cell death) compared to NRH alone (51% cell death), but no significant difference compared to paclitaxel alone (40% cell death). The OVCAR8 response was a bit more nuanced secondary to the sensitivity of this line to paclitaxel. A significant combination effect was noted in OVCAR8 with paclitaxel 20 nM + NRH (93% cell death) compared to NRH alone (51% cell death) and with paclitaxel alone (20 nM; 91% cell death). However, there was a high baseline cytotoxicity to paclitaxel alone, which made these data difficult to interpret.

The combination of carboplatin 1 µM + paclitaxel 2 nM (C/T) did not show significant additive cytotoxicity in any of the cell lines tested relative to C/T or NRH alone, (Figure 1D). While not statistically significant, in OVCAR8 cells there was a greater cytotoxic effect with the C/T + NRH combination than either NRH alone (51% cell death) or the C/T combination alone (24% cell death), though this did not reach statistical significance (*p* = 0.058).

**Figure 2:**
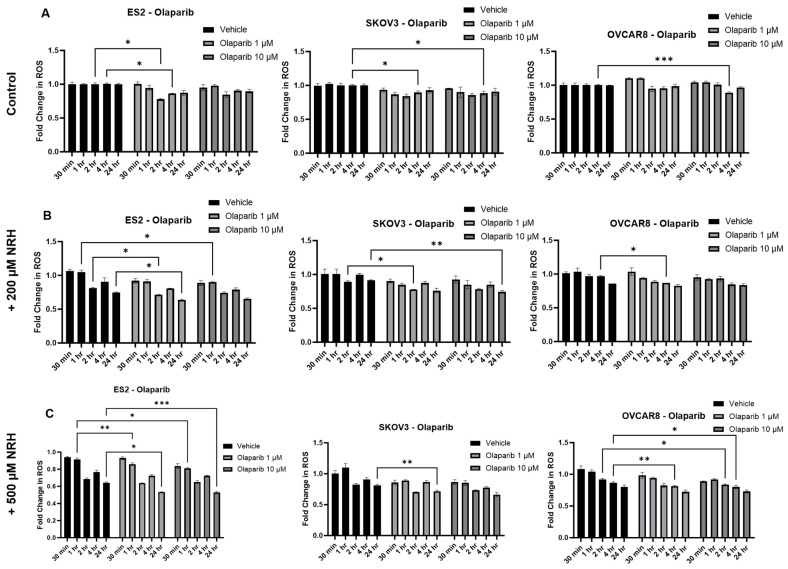
Cell death occurs independent of ROS generation. (**A**) EOC cell lines were treated over time up to 24 h with vehicle or in the presence of either 1 µM or 10 µM olaparib. (**B**) EOC cell lines were treated over time up to 24 h with vehicle or in the presence of either 1 µM or 10 µM olaparib in the presence of 200 µM NRH. (**C**) EOC cell lines were treated over time up to 24 h with vehicle or in the presence of either 1 µM or 10 µM olaparib in the presence of 500 µM NRH. * *p* < 0.05; ** *p* < 0.01; *** *p* < 0.001.

Olaparib was chosen for further study due to the marked increase in cytotoxicity when combined with NRH and because NAD+ is a substrate for PARP activity. As NAD+ concentration should directly impact PARP efficacy in DNA damage repair, we posit that NAD+ concentrations could modulate the cytotoxic impact of PARP inhibition. Effectively, if there is a low NAD+ concentration, the inhibition of PARP is unlikely to be effective, irrespective of the presence of a synthetically lethal genomic background. To better understand the etiology of the cytotoxic effect of the NRH/PARPi combination, we assessed ROS concentration in cell lines treated with NRH and the Olaparib/NRH combination. Previously, NRH was shown to increase ROS concomitantly with an increase in DNA damage in HepG3 liver cells [14]. We hypothesized that an increase in DNA damage would therefore increase PARP activity and thus, an increase in cellular ROS [8]. As shown in Figure 2, the cytotoxicity seen in Figure 1 appeared to be independent of ROS. In fact, compared with the control (Figure 2A), 200 µM NRH (Figure 2B) and 500 µM NRH (Figure 2C) showed a decrease in ROS over time up to 24 h and when combined with up to 10 µM olaparib for all cell lines tested. Therefore, ROS generation did not appear to be a primary driver of cell death in any of our lines, nor did modulation of NAD+ concentration alter this.

**Figure 3:**
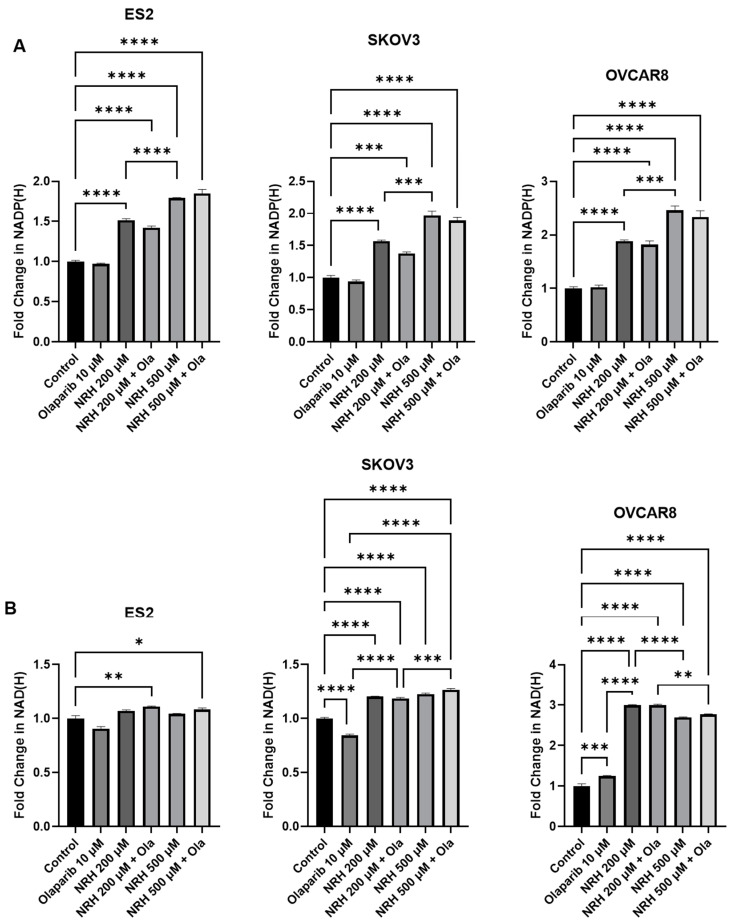
NRH supplementation induces an increase in NAD(P)(H). EOC cell lines were treated with 10 µM olaparib alone or in combination with 200 µM NRH and 500 µM NRH and analyzed for the generation of (**A**) NADP(H) and (**B**) NAD(H). * *p* < 0.05; ** *p* < 0.01; *** *p* < 0.001; **** *p* < 0.0001. (Ola = Olaparib).

As concentrations of NADP+ have been shown to modulate PARP inhibition efficacy in cancer [18], we assessed the levels of NADP(H) and NAD(H) in the presence of NRH and olaparib [13,15]. As shown in Figure 3A, NRH treatment resulted in a significant dose-dependent increase in NADP(H) from control, 200 µM NRH and 500 µM NRH. This effect was seen in all cell lines tested and was consistent in combination with olaparib 10 µM. Interestingly, olaparib alone did not alter NADP(H) levels. Additionally, a small yet significant NAD(H) increase in ES2 cells (Figure 3B) was only noted in combination groups. SKOV3 exhibited a decrease in NAD(H) in olaparib-treated cells, yet an increase in NAD(H) in NRH-treated cells. This change was noted to be dose-dependent in the combination groups from control, 200 µM NRH + olaparib, and 500 µM NRH + olaparib. OVCAR8-treated cells showed the largest increase in NAD(H) in NRH treated cells.

**Figure 4:**
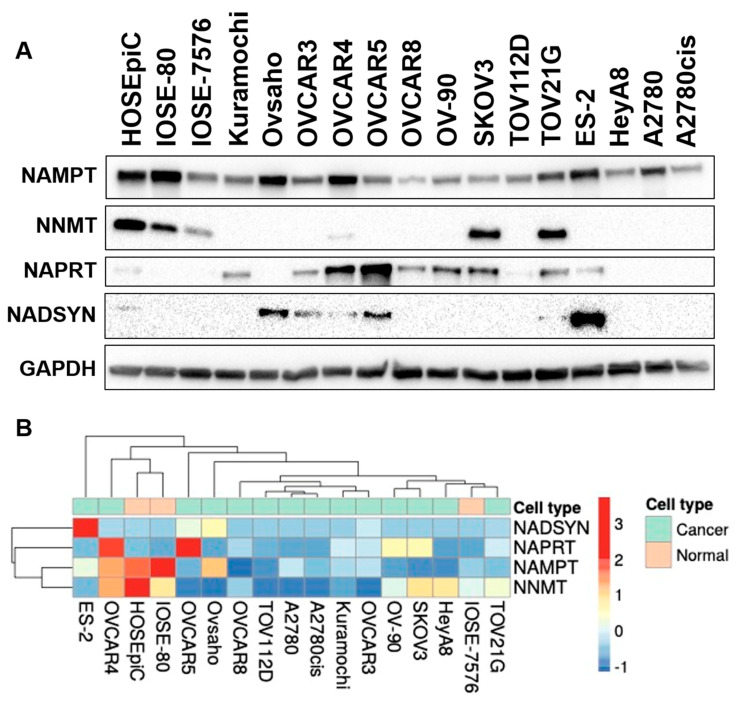
(**A**) Western blot analysis of key enzymes in the NAD+ metabolic pathway across multiple ovarian cell lines. (**B**) Quantification analysis of data shown in (**A**).

We then assessed the presence of key NAD+ biosynthetic pathway enzymes via western blot analysis. Of the cell lines used in this study, SKOV3 showed the highest expression of both nicotinate phosphoribosyltransferase (NAPRT) and nicotinamide n-methyltransferase (NNMT), ES2 showed the highest expression of nicotinamide phosphoribosyltransferase (NAMPT), and OVCAR8 showed the lowest expression of NAMPT (Figure 4A,B). ES2 cells showed the highest expression among the cell lines used in NAD synthase (NADSYN). Due to these differences, as well as the changes in NAD(P)(H) seen in NRH and olaparib treatments, we next sought to analyze metabolic activity during treatment.

**Figure 5:**
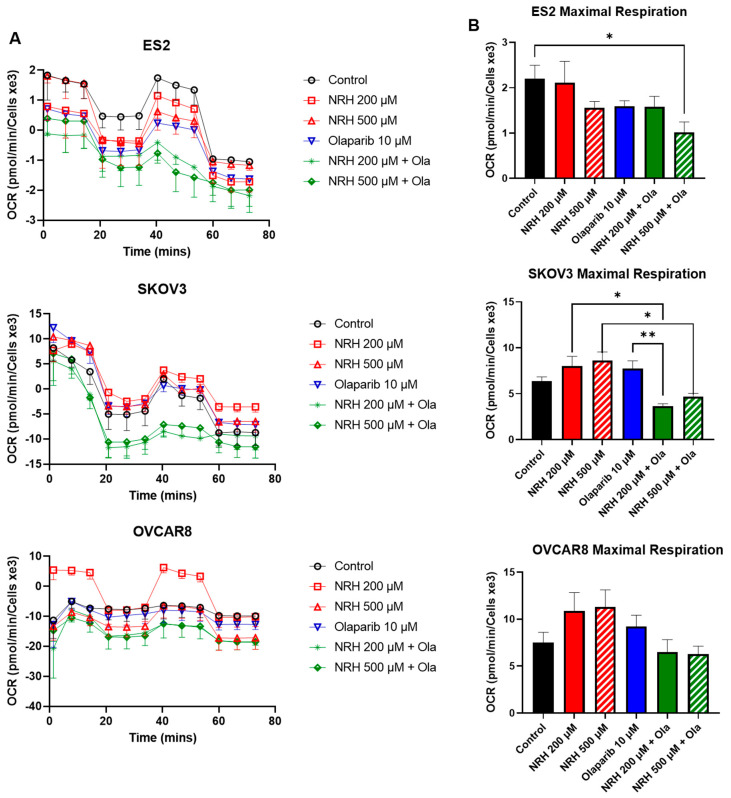
Mitochondrial respiration analysis of EOC cell lines. EOC cell lines were treated with 10 µM olaparib alone or in combination with 200 µM NRH and 500 µM NRH and analyzed for mitochondrial respiration. (**A**) Kinetic analysis of mitochondrial respiration of EOC cell lines over time. (**B**) Analysis of maximal mitochondrial respiration of EOC cell lines reveals distinct differences based on combination treatments. * *p* < 0.05; ** *p* < 0.01; (Ola = Olaparib) (OCR = oxygen consumption rate).

After treatment with the olaparib/NRH combination, a seahorse mitochondrial stress assay was used to analyze changes in mitochondrial respiration. As seen in Figure 5A,B, treatment with 200 µM NRH or 500 µM NRH showed no significant changes in either ES2 cells or SKOV3 cells. However, 200 µM NRH showed improved mitochondrial respiration compared to control in OVCAR8 cells. The addition of 10 µM olaparib to 200 µM and 500 µM NRH showed a reduction in mitochondrial respiration in both ES2 and SKOV3 cells (Figure 5A). Further analysis shows a significant reduction in maximal respiration in olaparib + 500 µM NRH in ES2, and both olaparib + 200 µM NRH and olaparib + 500 µM in SKOV3 cells (Figure 5B).

## 3. Discussion

NRH supplementation induced significant cytotoxicity in ovarian cancer cell lines in vitro. Our data were consistent, in this regard, to previous works demonstrating NRH supplementation inducing cell death in other cancer types [15,16]. This effect has not been observed in non-cancerous cells, suggesting, unsurprisingly, that the metabolic landscape of cancer is tenuous and this vulnerability an exploitable target for cancer therapy. NRH supplementation has been shown to be a tolerable and effective supplementation strategy for the increased production of NAD+ [16,19,20,21]. Given the relative dependence of DNA damage/repair and microtubule function on the NAD+ biosynthetic pathway, we sought to assess whether modulation of NAD+ concentration with NRH supplementation, combined with conventional treatment, enhances cytotoxicity in ovarian cancer where many of the primary mechanisms of disease response hinge on NAD+-dependent processes.

Not unexpectedly, given the intimate relationship of NAD+ with PARP activity, treatment of ovarian cancer cells with the PARP inhibitor, olaparib, supplemented with NRH, was shown to enhance cellular cytotoxicity across all cell lines tested. This supports our hypothesis that PARP inhibition efficacy is not only modulated by the presence of a synthetically lethal genomic landscape in tumors, but also by the metabolic landscape reflected in the concentration of its substrate, NAD+.

There was not a significantly enhanced effect when carboplatin was supplemented with NRH. The effects of paclitaxel were difficult to judge in this study, even at low doses, given the exquisite sensitivity of these cell lines to paclitaxel alone. Interestingly, the addition of NRH to high-dose carboplatin reduced the cytotoxicity in OVCAR8 cells. Coupled with the increase in NADP(H), this suggests an increase in carboplatin detoxification by glutathione as previously reported in lung cancer [22]. Previous data have shown that the mechanism of NRH supplementation-induced cytotoxicity is found in the generation of ROS [16]. However, our data indicate this is not the case in ovarian cancer cells. One explanation for this observation is that ovarian cancer cells have a larger antioxidant capacity to absorb the increase in ROS generated by the NRH supplementation-induced NAD+ boost. We then showed that, indeed, NRH supplementation did boost both NAD(H) and NADP(H) concentration in ovarian cancer cells with a greater effect on NADP(H). NADP(H) has been shown to induce both ROS generation as well as an antioxidant state in cells which would fit with our explanation [23]. Hence, the data presented in this study point to a brief antioxidant effect in the ovarian cancer cells studied. We hypothesized that this phenomenon may result in a diversion of bioenergetic enzymes towards ROS/redox management and away from DNA damage repair, which could explain the enhanced cytotoxicity to PARP inhibition.

The most responsive cell line, SKOV3, was shown to have an increase in NNMT as well as NAPRT in comparison to the others studied. As a metabolic enzyme, NNMT methylates nicotinamide generated from NAD+, and once methylated, N-methyl nicotinamide (1-MNAM) is terminally “stamped” for urinary excretion [24]. Therefore, an increase in NNMT should result in a decrease in the pool of free NAD+ available for biological processes. It is therefore possible that NRH supplementation, and hence NAD+ boosting, results in increasing the efficacy by which olaparib works, as it inhibits a DNA–PARP–NAD complex. Effects on the dose-dependent cytotoxic effects of paclitaxel were insignificant despite the addition of NRH and subsequent increases in NAD(P)(H). More work will be needed in regard to altered cellular metabolism and its effects on the mechanism of action of paclitaxel.

Interestingly, while NA leads to the production of NAD+ metabolically, we saw no significant cytotoxic activity associated with NA supplementation despite an increase in NAPRT, which is integral in the production of NAD+ from NA. This is consistent with prior work showing NRH as a much more effective NAD+-boosting agent than NA. However, it should also be noted that cells were grown under suggested ideal parameters without additional supplementation of nicotinamide or nicotinic acid, which can alter the expression of NAPRT and NAMPT. Regardless, the supplementation of NRH was shown to boost NAD(P)(H) in this study.

Finally, to better understand the cellular metabolic landscape in ovarian cancer cells supplemented with NRH, we performed an analysis of mitochondrial metabolic activity. We showed that olaparib with NRH supplementation reduced mitochondrial respiration efficiency in ovarian cancer cells when challenged. This supports our hypothesis that diversion of cellular resources to ROS management and terminal depletion of NAD+ stores leads to a breakdown in cellular respiration and ultimately cell death, though further work is needed to confirm this in animal models. This phenomenon could also be compounded by the conversion of NAD+ to NADP(H), leading to a further decrease in NAD+ stores. Additionally, NADP+ has previously been shown to act as an endogenous inhibitor to PARP, thereby sensitizing ovarian cancer cells to PARP inhibitor treatment both in vitro and in vivo [18]. This would further explain the combinatorial effects seen when olaparib is supplemented by NRH, whereby NAD+, not being used by PARP, is converted to methyl-NAM (specifically in the higher NNMT SKOV3 cell line), leading to the complete metabolic shutdown seen in Figure 5.

This study is the first, to our knowledge, that utilizes NRH as a supplement to enhance cytotoxicity in the treatment of ovarian cancer cells, both alone and in combination with standard treatments. We were able to show that the mechanism of cell death was a direct result of an increase in NAD(P)(H) and not directly related to ROS production. Rather, mitochondrial analysis revealed a decrease in mitochondrial respiration with combination treatments that would ultimately directly result in cell death. While previous studies have shown NRH supplementation in other cancer cell types with no cytotoxic effects on normal cells [16,21], no study has sought to include NRH supplementation as a dietary supplement in the treatment regimen for ovarian cancer. The data presented in these studies demonstrate the effects of NRH supplementation in an acute setting in vitro. Further pre-clinical research in chronic conditions and with animal models, in addition to prospective clinical trials, are needed to determine the safety and efficacy of combination treatments of NRH, in addition to standard treatments in ovarian cancer.

## 4. Materials and Methods

### 4.1. Cell Culture and Reagents

Ovarian cancer cell lines SKOV3 and ES2 were purchased from the American Type Culture Collection (ATCC) and grown according to the manufacturer’s recommendations. The NCI Frederick Cancer DCT Tumor Repository provided the OVCAR8 cell line. SKOV3 and ES2 cells were maintained in McCoy’s 5a medium (ThermoFisher, Waltham, MA, USA) supplemented with 10% fetal bovine serum (FBS) (ThermoFisher, Waltham, MA, USA), and OVCAR8 cells were maintained in RPMI1640 medium (Thermo Fisher Scientific, Waltham, MA, USA) supplemented with 10% FBS. Cells were confirmed to be free of mycoplasma contamination prior to the start of these studies and monitored on a regular basis. Commercially available chemotherapeutic agents (olaparib, carboplatin, and paclitaxel) were purchased from Selleck Chemicals (Houston, TX, USA) and stored according to the recommended procedures. NRH synthesis has been previously described [15,25].

### 4.2. Cytotoxicity

Cells were plated in 96-well black-wall, clear-bottom tissue culture plates at a concentration of 1000 cells per well and allowed to adhere overnight. Cells were then treated with carboplatin (C) at either 1 µM or 10 µM in H_2_O; olaparib (O) at either 1 µM or 10 µM in DMSO; paclitaxel (T) at either 2 nM or 20 nM in DMSO; a combination of carboplatin 1 µM + paclitaxel 2 nM (C/T); NRH at 500 µM in H_2_O; NA at 50 µM in H_2_O; in addition to combinations of carboplatin, olaparib, and paclitaxel with either NRH or NA. Treatments were allowed to incubate for 72 h at 37 °C. Cell cytotoxicity was measured with the CellTiter-Glo Assay (Promega, Madison, WI, USA) as per the manufacturer’s protocol. Luminescence reading was performed with the use of a Tecan (Maennedorf, Switzerland) plate reader. Dose-response curves and IC50 values were calculated on GraphPad Prism and reported as average ± standard error of the mean (SEM). GraphPad Prism version 10.0.2 for Windows, GraphPad Software, Boston, MA, USA, www.graphpad.com.

### 4.3. Reactive Oxygen Species Determination

Cells were plated in 96-well black-wall, clear-bottom tissue culture plates at a concentration of 5000 cells per well and allowed to adhere overnight. Cells were then treated with olaparib at either 1 µM or 10 µM alone or in combination with either 200 µM NRH or 500 µM NRH for 30 min, 1 h, 2 h, 4 h, and 24 h. Reactive oxygen species (ROS) was determined through the use of the fluorescent ROS indicator CM-H2DCFDA reagent (ThermoFisher, Waltham, MA, USA). Upon the completion of each time point, medium was removed from the wells and 2.5 µM reagent was added and incubated for 30 min at 37 °C. Fluorescence was analyzed with the use of a Tecan plate reader with an excitation wavelength of 495 nm and an emission wavelength of 525 nm. Data were plotted through the use of GraphPad Prism software as fold change over control and reported as average ± standard error of the mean (SEM).

### 4.4. NAD(P)(H) Assessment

Cells were plated in 96-well black-wall plates at 7500 cells per well and allowed to adhere overnight. Cells were then treated with olaparib (10 µM), NRH at either 200 µM or 500 µM, and a combination of either olaparib + NRH-200 µM or olaparib + NRH-500 µM, and allowed to incubate for a total of 4 h at 37 °C. NAD(P)(H) levels were analyzed through the use of the NAD/NADH-Glo assay and the NADP/NADPH-Glo assay (Promega) according to the manufacturer’s recommendations. Both assays converted either NAD+ or NADP+ to NADH and NADPH, respectively, and reported as NAD/NADH and NADP/NADPH, respectively, and reported as average ± standard error of the mean (SEM).

### 4.5. Western Blot

Cells were grown according to the manufacturer’s recommendations in 150 mm Petri dishes to ~80% confluency before being scraped and collected for cell lysis. Cell lysates (30 μg of protein per lane) were separated by polyacrylamide gel (10–15%) electrophoresis and transferred to nitrocellulose membranes. Membranes were then blocked with 5% milk in Tris-Buffered Saline (TBS) containing 1% Tween (TBS-T). Membranes were incubated overnight at 4 °C with the relevant primary antibody. The next day, membranes were washed with TBS-T and a HRP-secondary antibody was added for 1 h at room temperature on an orbital shaker. The membranes were then washed again with TBS-T three times prior to imaging. HRP-secondary antibodies were detected by incubating the membranes with WesternBright Sirius chemiluminescence.

### 4.6. Oxygen Consumption Analysis

Mito Stress Test Seahorse experiments were performed as previously described using a Seahorse XFe96 cell flux analyzer (Agilent Technologies, Tokyo, Japan) [26]. Ovarian cancer cells were plated at 5000 cells per well in a Seahorse cell culture 96-well microplate and allowed to adhere overnight at 37 °C. The sensor cartridge was hydrated at the time of cell plating and left overnight in a CO_2_-free incubator at 37 °C. The next day, cells were treated with olaparib (10 µM), NRH at either 200 µM or 500 µM, and a combination of either olaparib + NRH-200 µM or olaparib + NRH-500 µM and allowed to incubate for a total of 4 h at 37 °C. Injections for each assay were prepared according to the manufacturer’s instructions and loaded into the sensor cartridge. At the end of the incubation period, Seahorse medium with the required supplements was used to wash the treated cells and the plate was then placed in a CO_2_-free incubator for 30 min. During the 30 min incubation period, the sensor cartridge was placed in the XFe96 Seahorse analyzer for calibration (~20 min). At the end of the calibration and 30 min incubation period, the cell culture microplate was placed into the analyzer and Mito Stress Test was performed. Data were reported as average ± standard error of the mean (SEM).

### 4.7. Statistics

All assays were performed with a minimum of three biological replicates. All data were analyzed and plotted using GraphPad Prism software. For statistical analysis, comparison between two groups used a Student’s *T* test in order to test for statistical differences, whereas three or more groups were analyzed using a one-way ANOVA to test for statistical differences.

## Data Availability

The data generated in this study are available within this article.
